# Mechanisms of a Human Skeletal Myotonia Produced by Mutation in the C-Terminus of Na_V_1.4: Is Ca^2+^ Regulation Defective?

**DOI:** 10.1371/journal.pone.0081063

**Published:** 2013-12-06

**Authors:** Subrata Biswas, Deborah A. DiSilvestre, Peihong Dong, Gordon F. Tomaselli

**Affiliations:** Department of Medicine, Division of Cardiology, Johns Hopkins University, Baltimore, Maryland, United States of America; Indiana University School of Medicine, United States of America

## Abstract

Mutations in the cytoplasmic tail (CT) of voltage gated sodium channels cause a spectrum of inherited diseases of cellular excitability, yet to date only one mutation in the CT of the human skeletal muscle voltage gated sodium channel (hNa_V_1.4_F1705I_) has been linked to cold aggravated myotonia. The functional effects of altered regulation of hNa_V_1.4_F1705I_ are incompletely understood. The location of the hNa_V_1.4_F1705I_ in the CT prompted us to examine the role of Ca^2+^ and calmodulin (CaM) regulation in the manifestations of myotonia. To study Na channel related mechanisms of myotonia we exploited the differences in rat and human Na_V_1.4 channel regulation by Ca^2+^ and CaM. hNa_V_1.4_F1705I_ inactivation gating is Ca^2+^-sensitive compared to wild type hNa_V_1.4 which is Ca^2+^ insensitive and the mutant channel exhibits a depolarizing shift of the V_1/2_ of inactivation with CaM over expression. In contrast the same mutation in the rNa_V_1.4 channel background (rNa_V_1.4_F1698I_) eliminates Ca^2+^ sensitivity of gating without affecting the CaM over expression induced hyperpolarizing shift in steady-state inactivation. The differences in the Ca^2+^ sensitivity of gating between wild type and mutant human and rat Na_V_1.4 channels are in part mediated by a divergence in the amino acid sequence in the EF hand like (EFL) region of the CT. Thus the composition of the EFL region contributes to the species differences in Ca^2+^/CaM regulation of the mutant channels that produce myotonia. The myotonia mutation F1705I slows I_Na_ decay in a Ca^2+^-sensitive fashion. The combination of the altered voltage dependence and kinetics of I_Na_ decay contribute to the myotonic phenotype and may involve the Ca^2+^-sensing apparatus in the CT of Na_V_1.4.

## Introduction

**Figure 1:**
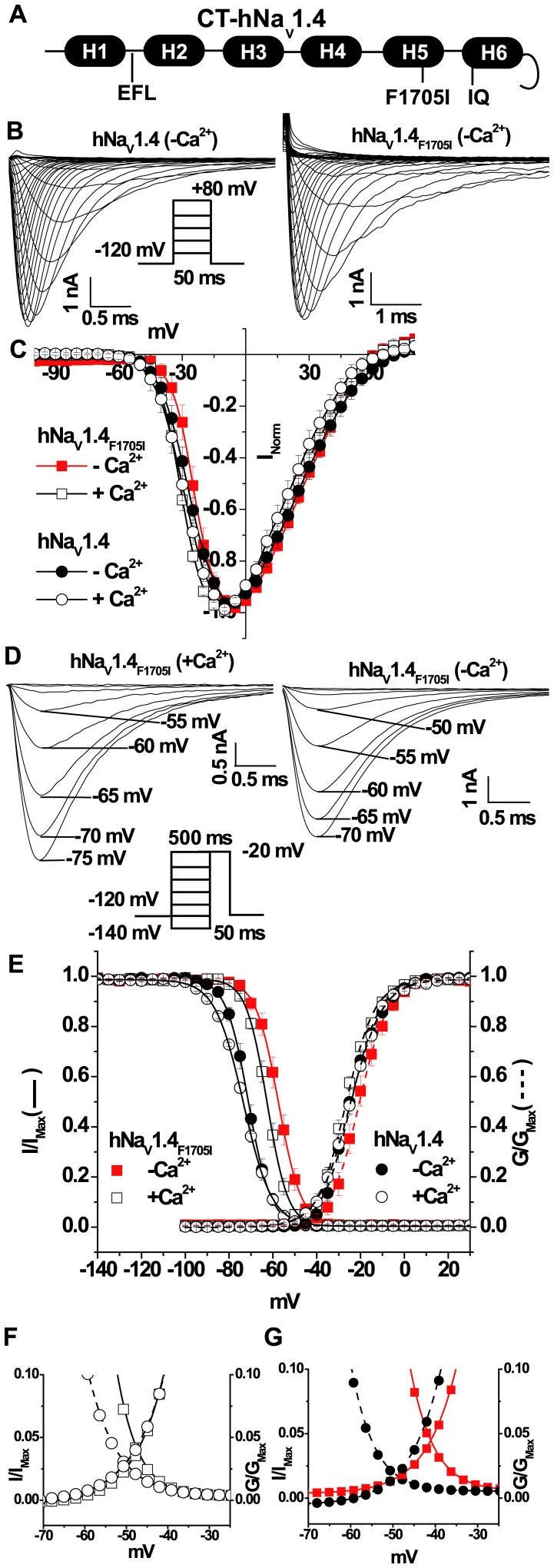
Ca^2+^-sensitivity of human Na_V_1.4_F1705I_ inactivation. (**A**) A schematic of the structured region of the C-terminus of hNa_V_1.4 between amino acids residues 1788 and 2040, the predicted helices are labeled H1–H6. The location of the EFL residues in and around H1 harbors species specific variations in the key Ca^2+^ sensing residues in hNa_V_1.4 (G1613S and A1636D) compared with the rat isoform. The CaM binding motif IQ in H6 and, the cold aggravated myotonia mutation F1705I (rat: F1698I) in H5 are illustrated. (**B**) Representative whole-cell currents through wild type and mutant hNa_V_1.4_F1705I_ channels expressed in HEK293 cells in [Ca^2+^]_i_ free conditions. Na^+^ currents were elicited by the protocol in the inset. (**C**) [Ca^2+^]_i_ does not alter the I–V relationship of hNa_V_1.4_F1705I_. (**D**) Representative steady-state inactivation currents from different holding potentials through mutant hNa_V_1.4_F1705I_ channels in the presence of 0.5 µM or absence of Ca^2+^. (**E**) Activation and steady-state inactivation curves of wild type and hNa_V_1.4_F1705I_ channels in the absence and presence of 0.5 µM intracellular Ca^2+^. The V_1/2_ of inactivation of hNa_V_1.4_F1705I_ is sensitive to [Ca^2+^]_i_ and significantly shifted in the hyperpolarizing direction in the presence of Ca^2+^ (p<0.005). The activation relationships are fitted with dotted lines, the V_1/2_ of activation are unaffected by change in [Ca^2+^]_i_. (**F**) and (**G**) illustrate the window currents through wild type and hNa_V_1.4_F1705I_ in presence (**F**) and absence (**G**) of intracellular Ca^2+^, respectively. Dotted lines represent the wild type channel. The symbols and color are the same in plots C, E, F, and G.

Precise and coordinated activity of skeletal muscle results from highly regulated signals generated by the orchestrated activities of different ion channels. A spectrum of muscle disorders are caused by the mutations in different ion channels [1]. A number of mutations in different regions of the human voltage-gated Na channel, hNa_V_1.4 have been reported to cause skeletal muscle disorders [2]. Mutations in the transmembrane domains and linker regions cause cold aggravated myotonia [3], [4], [5], [6] yet only one case of cold aggravated myotonia [7], has been linked to a mutation in the CT of the skeletal muscle sodium channel, hNa_V_1.4 (F1705I, Figure 1A) and functionally studied. The other mutation in the CT-Na_V_1.4, E1702K has been linked to paramyotonia congenita [8], but not functionally studied. Interestingly, another mutation Q1633E, causes potassium aggravated myotonia and is located in the EF hand like (EFL) region, that is the region in and around Helix 1, and the loop between Helix 1 and Helix 2 of the CT-hNa_V_1.4, and about 71 amino acids upstream of the E1705I mutation. Q1633E and F1705I have comparable electrophysiological effects including disruption of fast inactivation, slowed current decay, and a depolarized shift in the voltage dependence of availability [9]. hNa_V_1.4 mutations that cause myotonic disorders are associated with changes in the kinetics and voltage dependence of gating generally resulting in a gain-of-function.

There is substantial evidence that the CT of Na_V_ channels regulate the kinetics and voltage dependence of inactivation [10], [11], [12], [13]. Moreover, Ca^2+^ and CaM/CaM kinase (CaMK) distinctly modulate inactivation of different isoforms of Na_V_ channels through interaction with structural motifs in the CT although the mechanisms are not fully understood [10], [11], [14], [15], [16], [17], [18]. There is evidence, particularly in HEK cells, that over expression of CaM will alter Na_V_1.4 channel gating [10], [15], [16], and this is not unique to this channel but it also observed for other voltage dependent channels [15], [19], [20]. An EFL sequence [21], [22] in the CT of Na_V_1.5 and Na_V_1.1 has been shown to influence Ca^2+^ regulation of channel gating [14], [17], [18], [23], [24], [25]. Other acidic residues in the H1–H2 loop of the proximal C-terminus may affect Ca^2+^ sensitivity of channel gating as well [24], [26], and the NMR solution and crystal structures in this region reveals binding of Ca^2+^ [24] or Mg^2+^ [27]. In addition to the EFL, sites in the III–IV linker are involved in Ca^2+^/CaM mediated regulation of gating [28], [29], [30]. Similar Ca^2+^/CaM sites are also present in the CT of Na_V_1.4; however, their roles in channel regulation and relevance to disease causing mutations of the CT-Na_V_1.4 are not known. The goals of this study are to understand the pathogenesis of temperature sensitive myotonia caused by the F1705I mutation, and to use this naturally occurring mutation (and the species specific differences) to better understand the regulation of the Na_V_ channels by Ca^2+^. We demonstrate that the hNa_V_1.4_F1705I_ alters the voltage dependence of inactivation and the temperature sensitivity of current kinetics. In addition, key residues in the EFL alter the CaM and Ca^2+^ dependence of channel gating which may also contribute to the myotonia phenotype.

## Materials and Methods

### Plasmid Construction

The EYFP fused channel construct Na_V_1.4-EYFP was prepared as described previously [15]. The myotonia causing mutation in the rat CT, rNa_V_1.4_F1698I_, was made by site directed mutagenesis using primer pairs:

Forward (F): 5′CATGGAGGAGAAGATTATGGCAGCTAACCCTTC3′ and

Reverse (R): 5′GAAGGGTTAGCTGCCATAATCTTCTCCTCCATG3′. The human myotonia construct, hNa_V_1.4_F1705I,_ was made by site directed mutagenesis of the human hNa_V_1.4-EYFP construct using primer pairs:

Forward (F): 5′ CATGGAGGAGAAGATCATGGCAGCCAAC3′ and hNa_V_1.4_F1705I_ Reverse (R): 5′ GTTGGCTGCCATGA*TCT*TCTCCTCCATG3′. All the clones were sequence verified.

### Transfection of Cells

Approximately 0.75×10^6^ Human embryonic kidney cells (HEK293; American Type Culture Collection, Manassas, VA) were cultured in 6-well tissue culture dishes in DMEM supplemented with 10% FBS, L-glutamine (2 mmol/L), penicillin (100 U/mL), and streptomycin (10 mg/mL). The cells were co-transfected with plasmids encoding the β1 subunit and the appropriate fluorescently–tagged Na_V_1.4 and CaM variants: human or rat wild type Na_V_1.4-EYFP, hNa_V_1.4_F1705I_-EYFP, rNa_V_1.4_F1698I_-EYFP, hNa_V_1.4_F1705I+GA/SD_-EYFP and ECFP-CaM or ECFP-CaM_1234_ alone or in combination. Cells were transfected using Lipofectamine™ 2000 (Invitrogen) according to the manufacturer’s instructions and were studied 48 to 72 hours post-transfection. The total amount of DNA for all transfections was kept constant.

### Electrophysiology

HEK293 cells expressing wild type or mutant EYFP-tagged Na_V_1.4 channels were selected for recording. In some experiments, cells expressing both yellow and cyan fluorophores that were co-transfected with tagged Na_V_1.4 channel variants and CaM or CaM_1234_ were selected for patching. Cells were patch clamped with an Axopatch 200B patch-clamp amplifier using pipettes with tip resistances of 1–3 MΩ and typical series resistance compensation of >90% to minimize voltage clamp errors. Current recording was initiated 10 minutes after establishing whole-cell configuration, and currents were filtered at 5 kHz.

All the solutions used in this study were prepared as described previously [14], [17], [31], [32]. The bath solution contained (in mmol/L): 145 NaCl, 4 KCl, 1.8 CaCl_2_, 1 MgCl_2_, 10 glucose and 10 Na-HEPES (pH 7.4). The Ca^2+^ free patch pipette solution contained (in mmol/L): 10 NaF, 100 CsF, 20 CsCl_2_, 20 BAPTA, 0 CaCl_2_ and 10 HEPES, pH adjusted to 7.35 with CsOH. The 0.5 µmol/L Ca^2+^ patch pipette solution contained (in mmol/L): 10 NaF, 100 CsF, 20 CsCl_2_, 5 BAPTA, 4 CaCl_2_ and 10 HEPES, pH adjusted to 7.35 with CsOH. The osmolarity of the bath and pipette solutions were equalized using glucose. The free [Ca^2+^] in the solutions was estimated with WEBMAX Standard software (http://www.stanford.edu/~cpatton/webmaxcS.htm) and found to be about 0.5 µM. The free [Ca^2+^] in the solutions was verified by measurement using a Kwik-Tip Calcium ion-selective electrode (WPI) (Figure S1 and Table S1 in File S1). We studied the effect of intracellular Ca^2+^ on the voltage dependence of inactivation gating of rNa_V_1.4 with chloride substituted for fluoride in the pipette solution and found no significant difference in the Ca^2+^ sensitivity of gating (Figure S2 in File S1).

Standard two-pulse protocols were used to generate the steady-state inactivation curves. The voltage dependence of steady-state fast inactivation was studied using 500 ms inactivation pre-pulses over a voltage range from −140 to +30 mV in steps of 5 mV, followed by a 50 ms test pulse at −20 mV. Currents were normalized to the maximal current (I_max_) and fit to a Boltzmann function of the form (y = [(A_1_−A_2_)/(1+e^(x−x0)/dx)^)]+A_2_) to determine the membrane potential eliciting half-maximal inactivation (V_1/2_), where A_1_ and A_2_ are maximum and minimum availabilities, respectively, x0 is equivalent to V_1/2_, and dx represents the slope factor. Activation curves were generated from a family of 50 ms test pulses from −100 mV to +80 mV in 5 mV increments from a holding potential of −120 mV. Peak currents at each membrane potential normalized to I_max,_ were plotted to generate the I–V curves. Conductance (G) was calculated for peak current (I_Peak_) at each membrane potential (V_m_) using the equation (G = I_Peak_/(V_m_−V_Reversal_)). G was normalized to G_Max_ and fitted to a Boltzmann distribution to determine the membrane potential eliciting half-maximal activation (V_1/2_). Deactivation was assessed using tail currents elicited by a test pulse of 0.5 ms to +40 mV followed by repolarization to a family of voltages ranging from −180 to −50 mV. I_Na_ decay rates were assessed by fitting to a single exponential decay (Figure S3 in File S1). Recovery from inactivation was assessed by a standard two-pulse protocol with a first pulse duration of 30 ms and a second pulse of 30 ms to −20 mV with varying inter pulse intervals from 1 to 200 ms at −120 mV. Exponential functions of the form y = y0+Ae^−x/τ^ were fitted to recovery data to determine time constants (τ_rec_), where y0 is the offset and A is amplitude. Significance was assessed using unpaired student’s t-test (Microcal Origin, Microcal Software Inc. MA), and p<0.05 was considered significant.

## Results

### hNa_V_1.4_F1705I_ Alters Current Kinetics and Gating

A mutation (F1705I) in the structured portion of the CT of hNa_V_1.4 has been associated with cold aggravated myotonia (Figure 1A). We studied the effect of hNa_V_1.4_F1705I_ on channel function by transient expression in HEK293 cells at RT. Currents through wild type hNa_V_1.4 and hNa_V_1.4_F1705I_ channels were elicited by a family of depolarizing pulses ranging from −100 mV to +80 mV from a holding potential of −120 mV (Inset Figure 1B). The normalized peak current-voltage (I–V) relationships of wild type hNa_V_1.4 and hNa_V_1.4_F11705I_ were not different in 0.5 µM [Ca^2+^]_i_ mimicking the average intracellular [Ca^2+^] in muscle (Figure 1B and 1C). The voltage dependence (V_1/2_) of activation of the wild type and mutant channels did not differ in the presence of 0.5 µM [Ca^2+^]_i_ (Figure 1E and Table 1).

**Table 1:** Biophysical characteristics of Na_V_1.4 variants.

Channel/Mutant		Steady-state inactivation	Activation	τ_Rec_
		V_1/2_ (mV)	V_1/2_ (mV)	(ms)
**rNa_V_1.4**	+Ca^2+^	−64.2±0.1(7)	−29.0±0.3(6)	2.3±0.1(7)
	−Ca^2+^	−72±0.1(11)^a^	−31±1.5(13)	2.5±0.3(5)
**rNa_V_1.4**	CaM	−69.3±0.1(5)^a^	−30.6±0.2(6)	2.2±0.1(10)
**(0.5 µM Ca^2+^)**	CaM_1234_	−64.3±0.3(6)	−29.4±0.3(6)	2.6±0.1(5)
**rNa_V_1.4_F1698I_**	+Ca^2+^	−49.7±0.5(6)^a^	−16.6±1(6)^a^	1.7±0.1(5)
	−Ca^2+^	−49.7±0.8(9)	−15.3±1.3(7)	1.5±0.2(6)
**rNa_V_1.4_F1698I_**	CaM	−54.7±0.6(11)^b^	−17.9±0.7(9)	2.6±0.3(9)
**(0.5 µM Ca^2+^)**	CaM_1234_	−47.9±1.9(6)	−18.7±2(8)	3±0.3(5)
**rNa_V_1.4_SD/GA_**	+Ca^2+^	−70.0±0.2(8)^a^	−18.9±0.3(5)	2.3±0.1(8)
	−Ca^2+^	−70.4±0.2(6)	−19.0±0.3(6)	2.0±0.1(5)
**rNa_V_1.4_SD/GA_**	CaM	−62.9±0.1(6)^c^	−20.8±0.3(5)	2.9±0.1(6)
**(0.5 µM Ca^2+^)**	CaM_1234_	−64.1±0.1(8)^c^	−19.0±0.4(9)	2.3±0.1(9)
**rNa_V_1.4_F1698I+SD/GA_**	+Ca^2+^	−62.9±0.1(8)	−26±0.2(6)	2.1±0.1(8)
	−Ca^2+^	−57.3±0.1(5)^d^	−28.6±0.2(5)	2.0±0.1(5)
**rNa_V_1.4_F1698I+SD/GA_**	CaM	−51.5±0.1(6)^d^	−21.4±0.3(6)	2.3±0.1(7)
**(0.5 µM Ca^2+^)**	CaM_1234_	−52.9±0.1(5)^d^	−22.9±0.3(5)	2.1±0.1(6)
**hNa_V_1.4**	+Ca^2+^	−73.5±0.1(9)	−23±0.1(8)	2.4±0.1(6)
	−Ca^2+^	−71.2±0.1(7)	−24.4±0.3(5)	1.9±0.1(5)
**hNa_V_1.4**	CaM	−63.8±0.1(7)^e^	−15.4±0.2(6)^e^	2.8±0.1(5)
**(0.5 µM Ca^2+^)**	CaM_1234_	−65.1±0.1(6)^e^	−15.5±0.2(5)^e^	2.9±0.1(6)
**hNa_V_1.4**	CaM	−80.4±0.2(5)^f^	−15.5±0.2(5)	3.3±0.1(5)
**(0 Ca^2+^)**	CaM_1234_	−72.1±0.2(5)	−15.5±0.2(5)	2.4±0.1(5)
**hNa_V_1.4_F1705I_**	+Ca^2+^	−62.7±0.1(5)e1	−24.6±0.3(5)	1.7±0.1(5)
	−Ca^2+^	−57.5±0.1(7)^g^	−23±0.2(7)	3.1±0.1(5)
**hNa_V_1.4_F1705I_**	CaM	−56.8±0.1(5)^g^	−22.7±0.1(6)	2.7±0.1(5)
**(0.5 µM Ca^2+^)**	CaM_1234_	−52.4±0.1(5)^g^	−21.7±0.1(5)	2.2±0.1(5)
**hNa_V_1.4_F1705I_**	CaM	−61.7±0.1(5)	−22.9±0.1(6)	2.8±0.(3)
**(0 Ca^2+^)**	CaM_1234_	−60.4±0.5(5)	−21.7±0.1(5)	2.8±0.2(3)
**hNa_V_1.4_GS/AD_**	+Ca^2+^	−62.2±0.1(6)^e^	−19.8±0.2(6)	2.3±0.1(6)
	−Ca^2+^	−67.2±0.1(7)^h^	−16.0±0.2(8)	2.7±0.1(5)
**hNa_V_1.4_GS/AD_**	CaM	−70.2±0.1(5)h1	−20.5±0.2(6)	3.4±0.1(5)
**(0.5 µM Ca^2+^)**	CaM_1234_	−65.3±0.1(5)	−18±0.2(5)	3.3±0.2(5)
**hNa_V_1.4_F1705I+GS/AD_**	+Ca^2+^	−54.8±0.1(6)^g^	−23.3±0.3(6)	3.1±0.2(5)
	−Ca^2+^	−53.9±0.1(5)	−24.2±0.2(5)	2.4±0.1(5)
**hNa_V_1.4_F1705I+GS/AD_**	CaM	−59.3±0.1(5)^i^	−21.2±0.2(5)	2.7±0.1(5)
**(0.5 µM Ca^2+^)**	CaM_1234_	−56.8±0.1(7)	−20.3±0.3(9)	3.2±0.1(6)

Values: Mean±S.E(n); +Ca^2+^ = 0.5 µM Ca^2+^:

a vs. rNa_V_1.4 (0.5 µM Ca^2+^);

b vs. rNa_V_1.4_F1698I_ (0.5 µM Ca^2+^);

c vs. rNa_V_1.4_SD/GA_ (0.5 µM Ca^2+^);

d vs. rNa_V_1.4_F1698I_+_SD/GA_ (0.5 µM Ca^2+^);

e vs. hNa_V_1.4 (0.5 µM Ca^2+^);

e1 vs. hNa_V_1.4 (0.5 µM Ca^2+^);

f vs. hNa_V_1.4 (0 and 0.5 µM Ca^2+^);

g vs. hNa_V_1.4_F1705I_ (0.5 µM Ca^2+^);

h vs. hNa_V_1.4_GS/AD_ (0.5 µM Ca^2+^);

h1 vs. hNa_V_1.4_GS/AD_ (0.5 µM Ca^2+^);

i vs. hNa_V_1.4_F1705I+GS/AD_ (0.5 µM Ca^2+^).

The voltage dependence of steady-state inactivation was altered by hNa_V_1.4_F1705I_. Compared to the wild type channel, the V_1/2_ of the steady-state inactivation curve of hNa_V_1.4_F1705I_ was significantly shifted ∼+11 mV in 0.5 µM [Ca^2+^]_i_ (V_1/2_ hNa_V_1.4: −73.5±0.1 mV and V_1/2_ hNa_V_1.4_F1705I_: −62.7±0.1 mV, p<0.001; Figure 1E and Table 1). In the absence of a significant shift in the activation curve this produces a significant increase in the window current of hNa_V_1.4_F1705I_ compared to wild type hNa_V_1.4 in the presence or absence of [Ca^2+^]_i_ (Figures 1F and 1G). Thus, hNa_V_1.4_F1705I_ destabilizes steady-state inactivation and increases window current; however, the mechanism by which the mutation produces altered voltage dependence of gating is unknown. Given the importance of the CT of Na_V_ channels in the regulation of gating by Ca^2+^/CaM signaling we assessed whether hNa_V_1.4_F1705I_ interfered with channel modulation by Ca^2+^ or CaM.

### hNa_V_1.4_F1705I_ Alters Ca^2+^ Sensitivity

**Figure 2:**
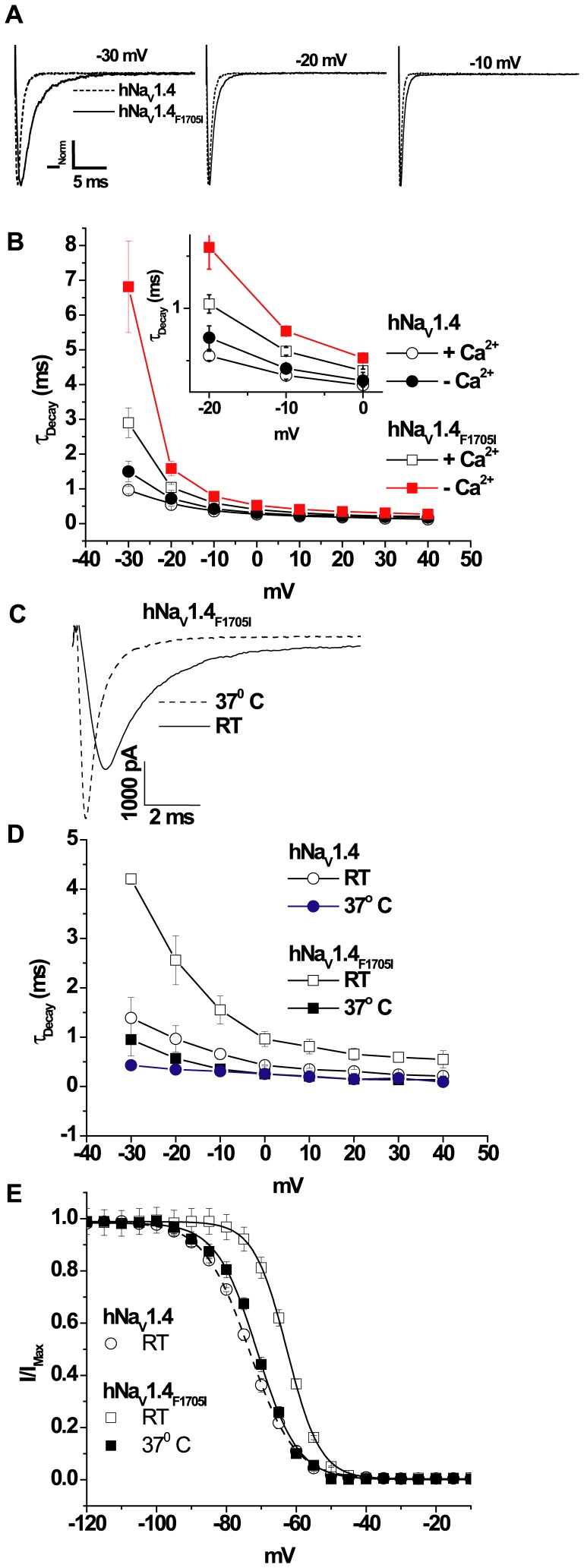
Current decay of myotonia mutant channels. The time constant of decay is altered in hNa_V_1.4_F1705I_ compared to wild type hNa_V_1.4 channels. (**A**) Superimposed normalized currents through wild type and hNa_V_1.4_F1705I_ channels at test pulses of −30, −20, and −10 mV exhibiting slowing of current decay in the mutant channel. (**B**) The time constants of current decay of hNa_V_1.4_F1705I_ channels are significantly different compared to wild type in the absence (p<0.05) or presence of 0.5 µM of Ca^2+^ (p<0.001). The inset is an expanded view −20 mV to 0 mV. (**C**) Raw current traces of hNa_V_1.4_F1705I_ channels at RT and at 37°C. Currents were measured at −20 mV, in the same cell at different temperatures with 0.5 µM of Ca^2+^ in the pipette. (**D**) Plot of the time constants of current decay of wild type and hNa_V_1.4_F1705I_ channels at RT and at 37°C in 0.5 µM of Ca^2+^. Paired measurements were made at RT and at 37°C. The current decay of hNa_V_1.4_F1705I_ is significantly different at 37°C compared to RT (p<0.05). (**E**) Plot of the steady-state inactivation of hNa_V_1.4_F1705I_ channels at 37°C in 0.5 µM of Ca^2+^. For comparison the steady-state inactivation of wild type and hNa_V_1.4_F1705I_ from Figure 1E are re-plotted. The symbols are the same in plots B, D and E.

Intracellular Ca^2+^ has been shown to regulate the cardiac isoform of Na_V_ channels, Na_V_1.5. It is not yet known if [Ca^2+^]_i_ similarly regulates skeletal muscle Na_V_1.4 channels and if mutations in the CT of the channel affect Ca^2+^ regulation. In order to understand Ca^2+^ mediated regulation of hNa_V_1.4 and hNa_V_1.4_F1705I_ we measured Na^+^ currents with 20 mM BAPTA in the pipette solution to create the Ca^2+^-free intracellular condition. The I–V relationships of wild type hNa_V_1.4 and hNa_V_1.4_F1705I_ are almost identical (Figure 1C); and the V_1/2_s of the activation (G−V) curves were unchanged in the absence of Ca^2+^ (Figure 1E and Table 1). The decay rate of wild type hNa_V_1.4 was insensitive to the intracellular [Ca^2+^]. The decay of hNa_V_1.4_F1705I_ currents is up to 2.5 times slower in the absence of Ca^2+^ compared to 0.5 µM [Ca^2+^]_i_ (Figures 2A, 2B and Table 2). The decay of hNa_V_1.4_F1705I_ was significantly slower than wild type hNa_V_1.4 at all negative voltages at RT in both low and 0.5 µM [Ca^2+^]_i_ (Figures 2A, 2B and Table 2). We studied the effect of hNa_V_1.4_F1705I_ on current decay at 37°C, as patients with the mutation do not exhibit myotonia at normal body temperature. In contrast to RT, at 37°C in 0.5 µM [Ca^2+^]_i_, the current decay of hNa_V_1.4_F1705I_ is markedly hastened (Figure 2C) and is not significantly different from wild type except at −30 mV (Figures 2C, and 2D, Table 2). At 37°C the V_1/2_ of steady-state inactivation of hNa_V_1.4_F1705I_ was significantly shifted in hyperpolarizing direction compared to RT (V_1/2_ at 37°C: −71.6±0.1 mV and V_1/2_ at RT: −62.7±0.1 mV, p<0.001; Figure 2E). I_Na_ decay, particularly at negative voltages, results from both inactivation and deactivation. We found no significant difference in the rates of deactivation at voltages between −180 and −100 mV (p<0.05; Figure S3 in File S1) similar to previous results [33]. However, at voltages from −90 mV to −60 mV, τ_Deactivation_ of hNa_V_1.4_F1705I_ in the Ca^2+^ free condition is significantly larger than in the wild type channel, with or without Ca^2+^ (p<0.05; Figure S3 in File S1).

**Table 2:** Na current decay time constants of hNa_V_1.4 and myotonia mutant hNa_V_1.4_F1705I._

Channel/Mutant		τ_Decay_ (ms)			
		−30 mV	−20 mV	−10 mV	0 mV
hNa_V_1.4	0.5 µM Ca^2+^	0.96±0.1(8)	0.54±0.05(8)	0.36±0.04(8)	0.26±0.02(8)
	Ca^2+^ free	1.5±0.3(5)	0.71±0.11(5)	0.42±0.05(5)	0.3±0.02(5)
	37°C (0.5 µM Ca^2+^)	0.43±0.01(5)	0.35±0.01(5)	0.3±0.02(5)	0.25±0.02(5)
hNa_V_1.4_F1705I_	0.5 µM Ca^2+^	2.9±0.4(5)#	1.04±0.09(5)#	0.59±0.03(5)#	0.4±0.01(5)#
	Ca^2+^ free	6.8±1.3(7)%	1.6±0.2(7)$	0.78±0.03(7)$	0.52±0.03(7)$
	37°C (0.5 µM Ca^2+^)	0.95±0.3(5)* ^@^	0.56±0.1(5)*	0.35±0.01(5)*	0.25±0.01(5)*

Values: Mean±S.E(n);

# vs. hNa_V_1.4 in 0.5 µM Ca^2+^;

% vs. hNa_V_1.4_F1705I_ (0.5 µM Ca^2+^);

$ vs. hNa_V_1.4_F1705I_ (0.5 µM Ca^2+^);

@vs. hNaV1.4 (37°C, 0.5 µM Ca2+);

* vs. hNa_V_1.4_F1705I_ (0.5 µM Ca^2+^).

Our previous work showed that the cardiac isoform hNa_V_1.5 is [Ca^2+^]_i_ sensitive, and lowering intracellular Ca^2+^ shifts the V_1/2_ of steady-state fast inactivation of hNa_V_1.5 in hyperpolarizing direction [14]. However, hNa_V_1.4 was insensitive to [Ca^2+^]_i_ and altering [Ca^2+^]_i_ does not significantly affect the voltage dependence of steady-state inactivation of wild type hNa_V_1.4 channels (Figure 1E). In contrast, the V_1/2_ of steady-state inactivation of hNa_V_1.4_F1705I_ was shifted significantly (∼5 mV) in the depolarizing direction in the absence of intracellular Ca^2+^ compared with the 0.5 µM [Ca^2+^]_i_ (Figures1D, 1E and Table 1). Thus hNa_V_1.4_F1705I_ exhibits Ca^2+^ sensitivity of steady-state inactivation distinct from wild type hNa_V_1.4 which is insensitive to changes in [Ca^2+^]_i_.

### hNa_V_1.4_F1705I_ Alters CaM Modulation

**Figure 3:**
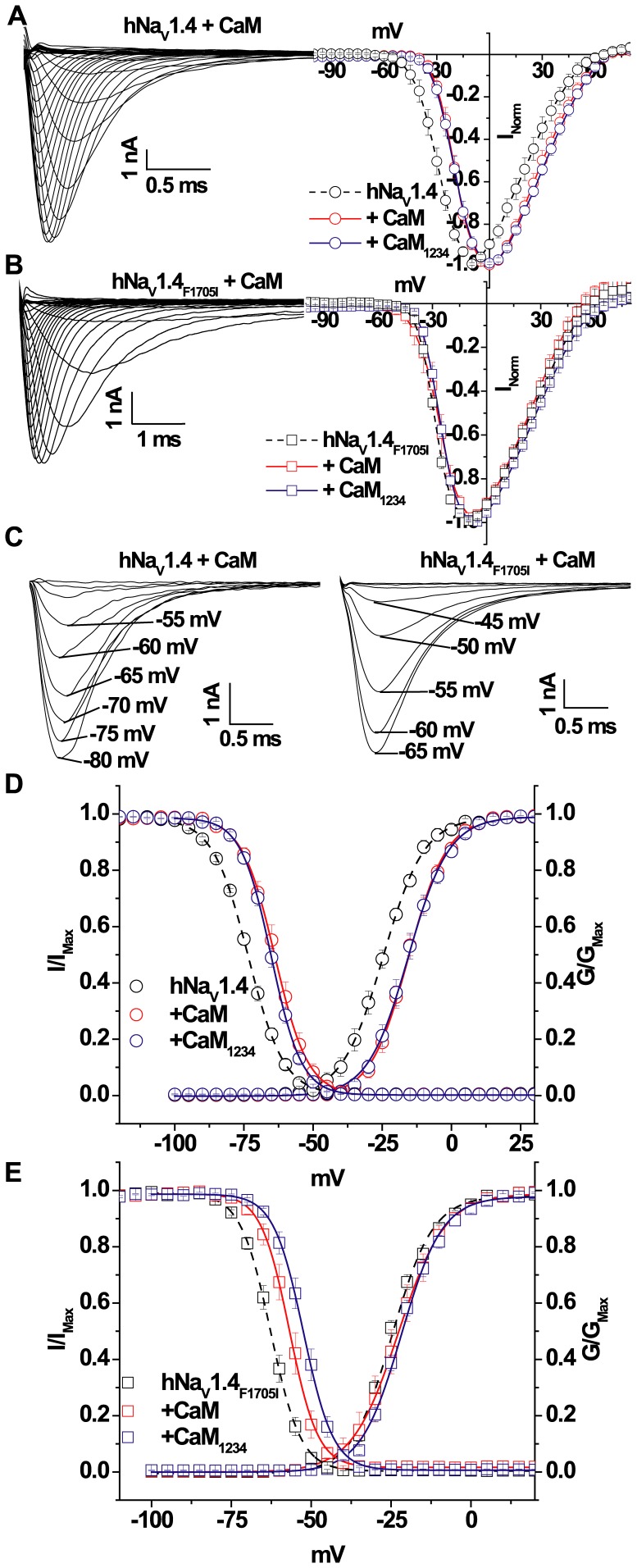
CaM-induced shift of inactivation in hNa_V_1.4 channels. Representative Na^+^ currents and I–V relationships for wild type (**A**) and hNa_V_1.4_F1705I_ channels (**B**) elicited by the same pulse protocol shown in the inset of Figure 1B. CaM and CaM_1234_ over expression significantly (p<0.05) shifts the wild type but not the hNa_V_1.4_F1705I_ I–V in the depolarizing direction. (**C**) Representative steady-state inactivation currents elicited from different holding potentials. (**D, E**) Plots of the steady-state inactivation and activation relationships of wild type hNa_V_1.4 (**D**) and hNa_V_1.4_F1705I_ channels (**E**). The solid lines are the fits to steady-state inactivation data with CaM and CaM_1234_ over expression. There is a significant (p<0.005) depolarizing shift of the inactivation curve by CaM and CaM_1234_ compared to the expression of hNa_V_1.4_F1705I_ alone. The symbols and colors are the same in all panels of the figure.

The F1705I mutation which is remote from the EFL region modifies the Ca^2+^ sensitivity of gating. F1705 is predicted to be in Helix 5 of the CT hNa_V_1.4 and is closer in the linear amino acid sequence to the CaM binding IQ motif in Helix 6 (Figure 1A). Previously we have shown that CaM binds to the IQ motif and shifts the voltage dependence of inactivation of rat Na_V_1.4 (rNa_V_1.4) and Ca^2+^ binding-deficient hNa_V_1.5 channels that are mutated in the EFL [10], [14], [15]. We tested the hypothesis that the large changes in Na^+^ current inactivation exhibited by hNa_V_1.4_F1705I_ compared to wild type hNa_V_1.4 are due to functional alterations in CaM interaction with the IQ motif. We examined the effects of CaM over expression on hNa_V_1.4_F1705I_ and wild type hNa_V_1.4 gating. As Ca^2+^ can bind to CaM and modulate channel gating, we also studied effect of Ca^2+^ binding deficient CaM or apo-CaM (CaM_1234_) to delineate direct effects of Ca^2+^ on the channel compared to Ca^2+^ effects through CaM. In contrast to the wild type rat Na_V_1.4 channel [15], over expression of both CaM and CaM_1234_ with wild type human Na_V_1.4 shifts the channel’s I–V relation in the depolarizing direction in 0.5 µM [Ca^2+^]_i_ (Figure 3A and Table 1). Co-expression of CaM and CaM_1234_ with hNa_V_1.4 significantly shifts the voltage dependence of activation in the depolarizing direction (Figure 3D). In contrast, neither the I–V relationships or voltage dependence of activation of hNa_V_1.4_F1705I_ were affected by co-expression with CaM or CaM_1234_ (Figures 3B, 3E and Table 1).

We previously demonstrated that CaM is tethered to the CT of Na_V_1.4, and CaM binding to the IQ motif shifts the steady-state inactivation of the rat isoform in a hyperpolarizing direction [10], [15] (Table 1). In contrast, co-expression of CaM with hNa_V_1.4 significantly shifts wild type channel availability in the depolarizing direction in 0.5 µM [Ca^2+^]_i_ (Figures 3C and D) and in the hyperpolarizing direction in the Ca^2+^ free intracellular condition, when compared with the absence of exogenous CaM (Table 1; p<0.05). CaM_1234_ over expression in 0.5 µM [Ca^2+^]_i_ shifts the V_1/2_ of inactivation of hNa_V_1.4 in the depolarizing direction compared to the absence of CaM_1234_ (Figure 3D; p<0.05). Upon removal of [Ca^2+^]_i_, the V_1/2_ of inactivation of hNa_V_1.4 is not affected by CaM_1234_ over expression (Table 1; p<0.05). Co-expression of CaM or CaM_1234_ with the mutant, hNa_V_1.4_F1705I_ also significantly shifts channel availability in the depolarizing direction compared to the absence of CaM co-expression (Figures 3C, 3E and Table 1; p<0.05). Similar to wild type, in Ca^2+^ free conditions, CaM or CaM_1234_ co-expression shifts V_1/2_ of steady-state inactivation in the hyperpolarizing direction (Table 1; p<0.05). Notably, in Ca^2+^ free conditions the CaM-induced hyperpolarizing shifts of steady-state inactivation of the wild type and mutant hNa_V_1.4 channels are in same direction as in the rat Na_V_1.4 channel.

We speculate that the difference in the voltage dependence of gating of wild type human and rat Na_V_1.4 channels when co-expressed with Ca^2+^-CaM/apo-CaM [15] may provide insights into the mechanisms of the Ca^2+^ modulation of channel function in the myotonia mutation and in wild type channels.

### Effect of rNa_V_1.4_F1698I_ on Ca^2+^/CaM Regulation of Channel Gating

**Figure 4:**
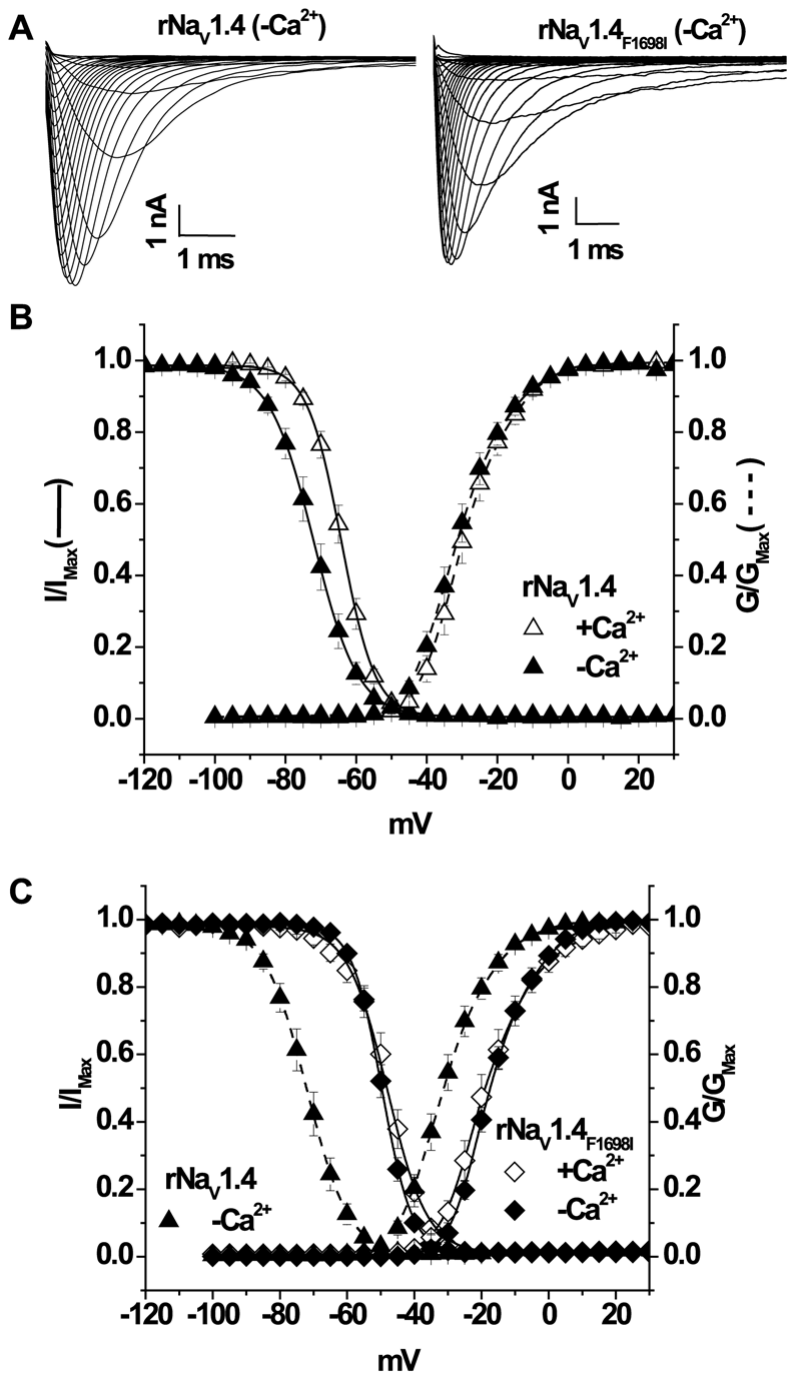
Ca^2+^ regulation of the rat Na_V_1.4. Whole-cell rNa_V_1.4 and rNa_V_1.4_F1698I_ expressed currents (**A**) are not affected by [Ca^2+^]_i_. (**B**) The voltage dependence of activation (dotted lines) and steady-state inactivation (solid lines) of rNa_V_1.4. (**C**) rNa_V_1.4_F1698I_ significantly (p<0.05) shifts the activation and inactivation curves in the depolarizing direction and eliminates the sensitivity of inactivation to changes in [Ca^2+^]_i_. The dotted lines represent the wild type channel in the absence of [Ca^2+^]_i_. The symbols are the same in plots B and C.

The wild type rat and human isoforms of Na_V_1.4 exhibit distinct differences in gating and regulation by Ca^2+^ and CaM. The orthologous mutation of hNa_V_1.4_F1705I_ in the rat is rNa_V_1.4_F1698I_. The activation curve of rNa_V_1.4_F1698I_ is significantly (p<0.05) shifted in the depolarizing direction (∼+12 mV) compared to wild type rNa_V_1.4 in 0.5 µM [Ca^2+^]_i_ (Figures 4A, 4B and Table 1). In contrast to rNa_V_1.4_F1698I_, no activation shift was seen with the F1705I mutation in human Na_V_1.4 (Figure 1E). Similar shifts in the activation curves of other Na_V_1.4 mutants that cause myotonia have previously been reported [34], [35], [36].

We next examined whether Ca^2+^ altered rNa_V_1.4_F1698I_ gating. The V_1/2_s of the activation of both the wild type rNa_V_1.4 and rNa_V_1.4_F1698I_ were not different in 0.5 µM Ca^2+^ compared with the absence of Ca^2+^ (Figures 4B, C and Table 1).

Unlike wild type rNa_V_1.4, the voltage dependence of the steady-state inactivation of rNa_V_1.4_F1698I_ was not affected by the [Ca^2+^]_i_ (Figures 4B, 4C and Table 1). Neither rNa_V_1.4 nor rNa_V_1.4_F1698I_ exhibit [Ca^2+^]_i_ sensitivity to recovery from inactivated states (Table 1). Thus the Ca^2+^ sensitivity of steady-state inactivation of rNa_V_1.4 was eliminated with a mutation remote in the linear amino acid sequence from the EFL motif. Although the rNa_V_1.4_F1698I_ results in loss of Ca^2+^ sensitivity of the channel, it also dramatically affects steady-state inactivation of the channel producing approximately ∼+15 mV shift of the V_1/2_ compared to the wild type rNa_V_1.4 in presence of 0.5 µM Ca^2+^ (Figures 4B, 4C and Table 1; p<0.05).

### IQ-CaM Interaction is Unaffected by rNa_V_1.4_F1698I_

**Figure 5:**
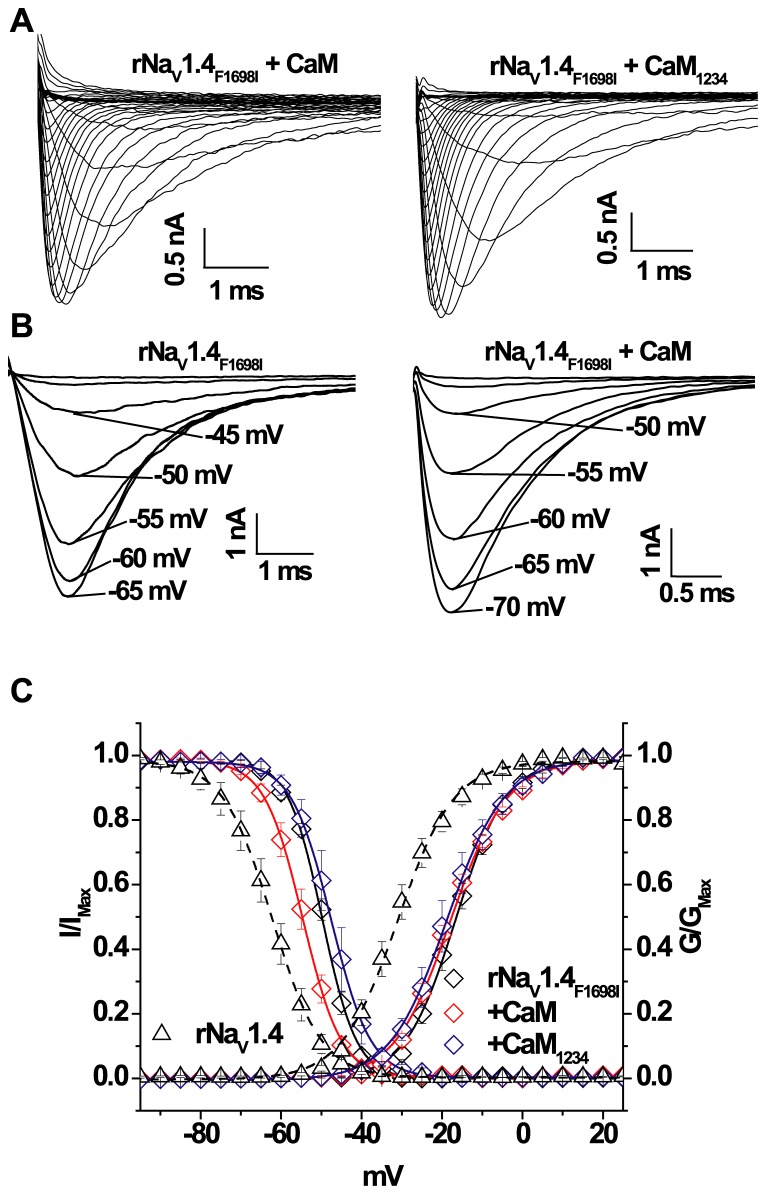
CaM shifts inactivation of rat Na_V_1.4_F1698I_. (**A**) Representative families of rNa_V_1.4_F1698I_ activation currents co-expressed with either CaM or CaM_1234_. (**B**) Representative steady-state inactivation currents elicited from different holding potentials through rNa_V_1.4_F1698I_ channels in presence and absence of CaM over expression. (**C**) Activation and steady-state inactivation relationships. The solid lines are the fits to steady-state inactivation data of rNa_V_1.4_F1698I_ with CaM and CaM_1234_ over expression. There is a significant (p<0.05) shift of the inactivation curve by CaM compared to the expression of rNa_V_1.4_F1698I_ alone. Over expression of CaM_1234_ has no significant effect compared with the absence of CaM over expression. In contrast, the V_1/2_ of activation of rNa_V_1.4_F1698I_, is not changed by co-expression of CaM or CaM_1234_. The dotted lines in panel (**C**) represent wild type channel in 0.5 µM Ca^2+^.

The significant alteration in Na^+^ current properties demonstrated by rNa_V_1.4_F1698I_ suggests the possibility of a disruption of CaM interaction with the IQ motif which is in vicinity of F1698. CaM or CaM_1234_ co-expression does not alter the current kinetics (Figure 5A) or the voltage dependence of activation of rNa_V_1.4_F1698I_ (Figure 5C). Similarly, CaM and CaM_1234_ do not affect the activation of wild type rNav1.4 (Table 1). However, co-expression of CaM with rNa_V_1.4_F1698I_ significantly shifts channel availability in the hyperpolarizing direction compared to the absence of CaM co-expression (Figures 5B and 5C; p<0.05). CaM_1234_ has no effect on the voltage dependence of steady-state inactivation in 0.5 µM [Ca^2+^]_i_ (Figure 5C). CaM and CaM_1234_ also have similar effects on wild type rNav1.4 availability (Table 1). The time constants of recovery from inactivated states of rNa_V_1.4_F1698I_ are unaffected by over expression of CaM or CaM_1234_ (Table 1). In both species the orthologous myotonia mutations in the CT affect the Ca^2+^ regulation of channel gating. In the rat channel rNa_V_1.4_F1698I_ abolished the Ca^2+^ sensitivity observed in the wild type rNa_V_1.4. In contrast, hNa_V_1.4_F1705I_ imparts sensitivity to Ca^2+^ which is absent in wild type hNa_V_1.4. We postulated that differences in the EFL region of the two channels contribute to the difference in Ca^2+^ response of the orthologous myotonia mutations in the human and rat channels.

### EFL Residues Mediate the Differences in Ca^2+^ Regulation

**Figure 6:**
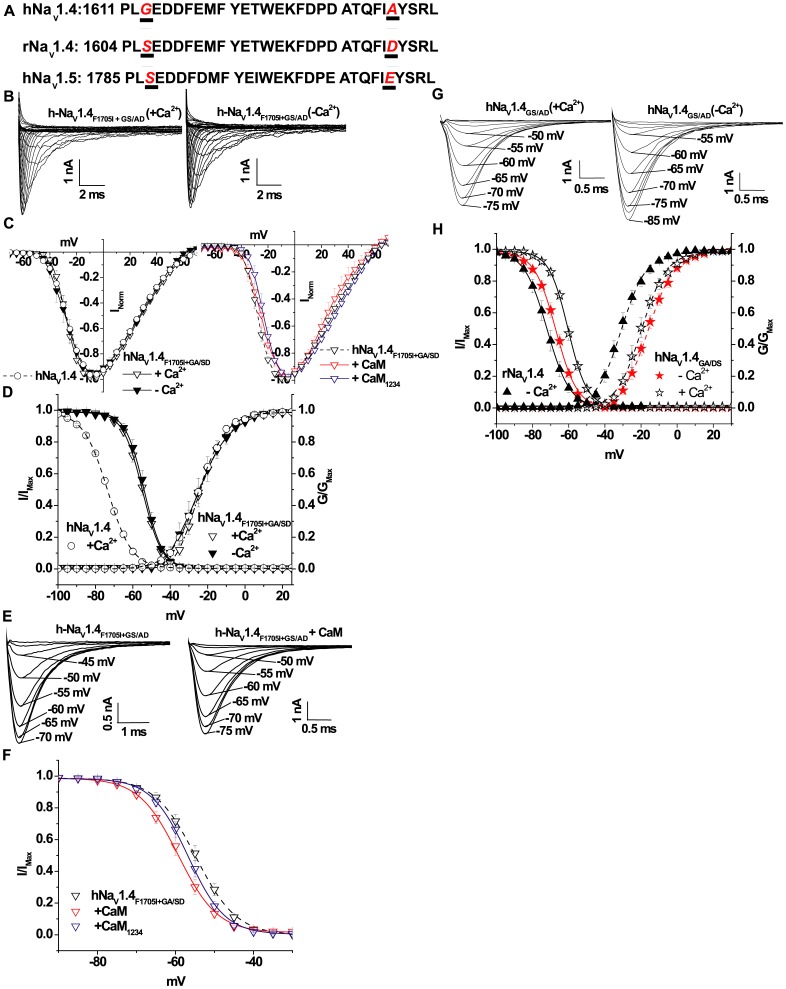
Exchange of human and rat EFL residues in hNa_V_1.4. (**A**) Amino acid sequence alignment of the proximal CT of rat and human wild type Na_V_1.4 channel and hNa_V_1.5 demonstrate the similarity of rNa_V_1.4 and hNa_V_1.5 at key positions in the EFL. In hNa_V_1.4_F1705I+GA/SD_ residues G1613S and A1636D are substituted in the human channel hNa_V_1.4_F1705I_ to match the corresponding residues of rNa_V_1.4. (**B**) Representative families of hNa_V_1.4_F1705I+GA/SD_ activation currents in the presence and absence of [Ca^2+^]_i_. (**C**) Normalized I–V relationships hNa_V_1.4_F1705I+GA/SD_ are not affected by altered [Ca^2+^]_i_ or CaM and CaM_1234_ over expression. (**D**) The steady-state inactivation of hNa_V_1.4_F1705I+GA/SD_ channels are not sensitive to changes in [Ca^2+^]_i_. The dotted lines in panel (**D**) represent hNa_V_1.4 in 0.5 µM Ca^2+^. (**E**) Representative steady-state inactivation currents elicited from different holding potentials through hNa_V_1.4_F1705I+GA/SD_ channels in the presence and absence of CaM over expression. (**F**) There is a significant (p<0.004) hyperpolarizing shift of the inactivation curve by CaM over expression compared to the expression of hNa_V_1.4_F1705I+GA/SD_ (in dotted line) alone. Over expression of CaM_1234_ has no significant effect compared with the absence of CaM over expression. (**G**) Representative steady-state inactivation currents elicited from different holding potentials through hNa_V_1.4_GA/SD_ channels in the presence and absence of [Ca^2+^]_i_. (**H**) Steady-state inactivation of hNa_V_1.4_GA/SD_ channel exhibited sensitivity to changes in [Ca^2+^]_i_ similar to the wild type rat channel.

We compared the amino acid sequences in the CT of the rat and human Na_V_1.4 channels in the EFL and IQ motifs. In the region that includes the EFL (Figure 1A), the amino acids at positions 1613 and 1636 in hNa_V_1.4 are glycine (G) and alanine (A), and the corresponding residues in rNa_V_1.4 are serine (S) and aspartic acid (D) respectively (Figure 6A). Position 1613 is within the predicted EFL region whereas residue 1636 is outside the EFL in H1–H2 loop region. We tested the hypothesis that hNa_V_1.4_F1705I_ altered channel gating by disruption of Ca^2+^ sensing through the EFL motif. We generated a triple mutant human channel, containing F1705I and replacing the non-conserved residues in the human EFL with the corresponding amino acids from the rat sequence hNa_V_1.4_F1705I,G1613S,A1636D_ (hNa_V_1.4_F1705I+GS/AD_). hNa_V_1.4_F1705I+GS/AD_ and hNa_V_1.4 have comparable peak current I–V relationships, and in both cases the voltage dependences of activation were not altered by the absence or presence of 0.5 µM Ca^2+^ (Figures 6B, 6C and Table 1). Similar to rNa_V_1.4_F1698I_, steady-state inactivation of hNa_V_1.4_F1705I+GS/AD_ was insensitive to changes in the [Ca^2+^]_i_ (Figure 6D and Table 1). Thus key residues in the EFL region contributed to the species differences in Ca^2+^ sensitivity of mutations in Na_V_1.4 that produce myotonia.

Remarkably hNa_V_1.4_F1705I+GS/AD_ exhibits CaM regulation that recapitulates that of rNa_V_1.4_F1698I_. Over expression of CaM with hNa_V_1.4_F1705I+GS/AD_ produces a hyperpolarizing shift in V_1/2_ of steady-state inactivation compared to the mutant channel in the absence of CaM over expression (Figures 6E, 6F and Table 1; p<0.05). Similar to rNa_V_1.4_F1698I_, CaM_1234_ over expression with hNa_V_1.4_F1705I+GS/AD_ does not alter the voltage dependence of steady-state inactivation compared to the mutant channel without CaM_1234_ over expression (Figure 6F and Table 1). The orthologous myotonia mutation on the rat channel background exhibits changes in Ca^2+^/CaM regulation that are completely different from that of hNa_V_1.4_F1705I_. However substituting the EFL residues present in the rat channel into the hNa_V_1.4_F1705I_ background is sufficient to recapitulate the Ca^2+^/CaM regulation exhibited by rNa_V_1.4_F1698I_ (Figures 6 and Table 1). This holds true for wild type channels, as mutation of the non-conserved amino acids of the wild type hNa_V_1.4 EFL to match that of the rat sequence (G1613S and A1636D) reestablished Ca^2+^ and CaM sensitivity of inactivation gating mimicking that of wild type rat Na_V_1.4 (Figures 6G and 6H, Table 1). Additionally, changing the EFL residues (G and A) from human channel into the wild type rat Na_V_1.4 (rNa_V_1.4_SG/DA_) or mutant rNa_V_1.4_F1698I_ (rNa_V_1.4_F1698I+SG/DA_) background restored Ca^2+^/CaM regulation similar to that displayed by hNa_V_1.4 or hNa_V_1.4_F1705I_ respectively (Table 1). Thus the differences in the key amino acids in the EFL region are associated with species-specific differences in Ca^2+^/CaM regulation of the wild type and mutant, human and rat Na_V_1.4 channels.

## Discussion

Myotonic mutations in hNa_V_1.4 have been reported to affect Na^+^ current inactivation properties. Wu *et al*. demonstrated a destabilization of fast inactivation without significant changes in activation or slow inactivation by the F1705I mutation [7]. However, the mechanism of the change in gating is uncertain as is the role of alteration of Ca^2+^ or CaM modulation of the function of the mutant channel. We have demonstrated that the CT myotonia mutation, hNa_V_1.4_F1705I_ slows the I_Na_ decay, depolarizes the voltage dependence of inactivation and augments the window current, effects that may contribute to the cold-induced myotonic phenotype. Notably, hNa_V_1.4_F1705I_ imparts intracellular Ca^2+^ sensitivity to inactivation gating, a feature that is distinct from the wild type hNa_V_1.4. However, the direction of the shift of steady-state inactivation of hNa_V_1.4_F1705I_ channels is opposite to the direction of the Ca^2+^-induced shift observed with cardiac channel, hNa_V_1.5 [14]. Our data suggest that two key residues in the EFL (Figure 6, Table 1) modify gating of wild type hNa_V_1.4 and the myotonia mutant hNa_V_1.4_F1705I_.

Sodium channel mediated myotonia is characterized electrophysiologically by a delay in inactivation that predisposes to repetitive depolarization and contraction of skeletal muscle after a brief stimulus. Previous studies suggested that a defect in fast inactivation, as in the CT mutant F1705I mutation, was sufficient to produce myotonia [7], [37]. Our data shows, that along with a depolarizing shift in steady-state inactivation and slowed I_Na_ decay, reduced intracellular Ca^2+^ levels exaggerate both the depolarizing shift of inactivation (Figures 1D, 1E and 1G) and the slowing of I_Na_ decay of the current (Figures 2A and 2B). It appears that the largest effect of the F1705I mutation is on the voltage dependence and temperature sensitivity of gating but our data suggest that altered [Ca^2+^]_i_ sensitivity of the mutant channel potentiates symptoms of myotonia at low temperature. The experiments were designed to test the effects of Ca^2+^ on channel gating and we explored the extremes of the range of concentrations. We do not believe that bulk Ca^2+^ levels ever reach these levels but sub cellular distribution of ion concentrations are heterogeneous and local changes in Ca^2+^ in this range are feasible [38].

Slowing of I_Na_ decay, increased window current, altered voltage dependence of inactivation, along with disruption of Ca^2+^ sensitivity of hNa_V_1.4_F1705I_ could explain myotonia, but it is not clear what triggers cold-induced symptoms or why these patients are free from symptoms at normal body temperature. Our study indicates hastening of I_Na_ decay of hNa_V_1.4_F1705I_ at normal body temperature eliminates current changes that are associated with the myotonic phenotype. At 37°C, the I_Na_ decay of hNa_V_1.4_F1705I_ (at −30 mV) is ∼5 times faster than at RT and is nearly identical to the decay rate of wild type hNa_V_1.4 current at 37°C (Figures 2C and 2D). Additionally, at 37°C, V_1/2_ of steady-state inactivation of hNa_V_1.4_F1705I_ significantly shifted ∼10 mV in hyperpolarizing direction compared to RT, and is comparable to V_1/2_ of wild type channel at RT (Figure 2E). The temperature dependent shift we observed in the human mutant channel is more than the previously reported ∼3 mV temperature dependent shift in steady-state inactivation of native rat skeletal muscle Na channels [39]. Rescue of I_Na_ decay and steady-state inactivation at 37°C could explain why the patients with the F1705I mutation are free from myotonia at normal body temperature. Slowing of deactivation of hNa_V_1.4 may cause sustained skeletal muscle contraction by delaying repolarization thereby prolonging action potential duration [34]. Differential temperature-induced changes in mutant channel gating; particularly slowing of deactivation resulting in persistent membrane depolarization has been proposed for some hNa_V_1.4 mutations causing myotonia [40]; however, the F1705I mutation does not alter deactivation [33]. Additionally, it has been suggested that at normal temperatures more wild type channels are activated compared to mutant channels and with lowering of temperature mutant channels dominate membrane excitability [41]. Alternatively changes in temperature may be associated with changes in intracellular [Ca^2+^] [42] that exaggerate the inactivation gating defects of the mutant channel. We speculate that temperature sensitive changes in intracellular Ca^2+^ could alter cold aggravated myotonia in F1705I mutant muscle.

The biophysical effects of hNa_V_1.4_F1705I_ and the orthologous rat mutation rNa_V_1.4_F1698I_ are consistent with a key role for the CT in inactivation gating. Residues in and around the EFL motif [21], [22] of the CT of hNa_V_1.5 and Na_V_1.1 regulate the Ca^2+^ sensitivity of channel gating [14], [17], [18], [23], [24], [25], which may occur at low levels of free [Ca^2+^] in the cell [43]. In addition to the EFL, sites in the III–IV linker are involved in Ca^2+^/CaM mediated regulation of gating [28], [29]. We exploited the differences in the EFL sequences and Ca^2+^ sensitivity of inactivation gating of the human and rat orthologous Na_V_1.4 channels to better understand the mechanisms of Ca^2+^ modulation of wild type and mutant channels. Inactivation gating of hNa_V_1.4_F1705I_ is sensitive to changes in intracellular [Ca^2+^], in contrast wild type hNa_V_1.4 exhibits no such sensitivity to Ca^2+^. The inverse is true for the wild type and mutant rat isoforms of Na_V_1.4. Mutating the non-conserved amino acids of the hNa_V_1.4 EFL region to match that of the rat sequence (G1613S and A1636D), abolishes the Ca^2+^ sensitivity of hNa_V_1.4_F1705I+GS/AD_ mimicking that of rNa_V_1.4_F1698I_. Similarly, mutating the non-conserved amino acids of the wild type hNa_V_1.4 EFL region to match that of the rat sequence (G1613S and A1636D) restores the Ca^2+^ sensitivity of the channel mimicking that of wild type rat Na_V_1.4 (Figures 6G, 6H, Table 1). To our knowledge, this is the first report of a mutation in the CT remote from the EFL region that abolishes Ca^2+^ sensitivity of gating of Na_V_1.4 channels; however, it is not clear that the rNa_V_1.4_F1698I_ or hNa_V_1.4_F1705I_ has compromised channel gating through a long range conformational effect on the EFL motif. Notably, both rat and human myotonic mutant channels dramatically shift the voltage dependence of inactivation gating in the depolarizing direction. A similar depolarizing shift in inactivation due to a mutation in the CT hNa_V_1.4 (Q1633E) has been reported, implicating the EFL region of the CT in inactivation gating [9]. Thus it may be that these mutants introduce a significant local structural change in the predicted H5 which in turn influences the neighboring H4 and alters helical interactions in the EFL leading to a disruption of Ca^2+^ sensing. Hydrophobic helical interactions in the cardiac Na_V_1.5 EFL, particularly H1–H4, appear to stabilize the structure of the proximal CT of the channel which is postulated to be a prerequisite for durable inactivation [44]. Although most of the mutations at the hydrophobic interfaces of the EFL helices shift inactivation in the hyperpolarizing direction, there are important exceptions to this generalization [44].

The mechanism of the Ca^2+^ regulation of gating by residues in the CT is not fully understood and the role of direct Ca^2+^ binding to this region is debated. In fact the crystal structure of a ternary complex of the CT-hNa_V_1.5, CaM and FHF fails to show Ca^2+^ in the EFL region [27], [30]. The structure needs to be viewed in the context of functional studies of the intact channel where mutations of the EFL residues consistently alter the Ca^2+^ sensitivity of hNa_V_1.5 gating [14], [17], [18], [24], [45]. This does not prove that Ca^2+^ is binding to this region; an alternative is that the mutation(s) produce an allosteric change that alters Ca^2+^ sensitivity of gating.

It is interesting that this myotonic mutation in H5 does not affect CaM regulation of the channel. This finding is consistent with previous observations [15] that CaM is an integral part of Na_V_1.4 and it remains tethered to the mutant channels in all conformations. An alternative explanation is that the IQ-containing H6 interacts with the EFL [24], [45] bringing the H5 into physical proximity of the EFL. Thus mutations such as rNa_V_1.4_F1698I_ or hNa_V_1.4_F1705I_ in H5 could alter the conformation and disrupt Ca^2+^-sensing but this would have to occur without altering the interaction between CaM and the IQ as myotonic mutant channels of both the species are normally regulated by over expression of CaM (Table 1).

**Figure 7:**
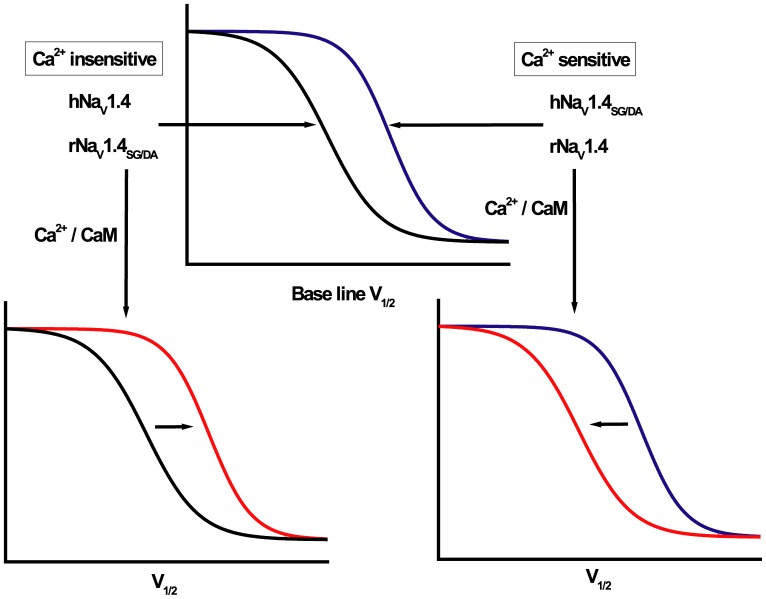
Ca^2+^ sensitivity, inactivation gating and calmodulation. This schematic illustrates the relationship between the Ca^2+^ sensitivity of inactivation gating and the effect of CaM over expression. Channel variants that exhibit shifts in the voltage dependence of inactivation as a function of changes in intracellular [Ca^2+^] exhibit a depolarized V_1/2_ compared with variants insensitive to Ca^2+^. The Ca^2+^ sensitivity is associated with the direction of the gating shift induced by CaM over expression. Residues in the EFL are key determinants of the Ca^2+^ sensitivity and effect of CaM over expression on inactivation gating. The red curves in plots indicate steady-state inactivation in the presence of CaM.

It is clear from our study that intracellular Ca^2+^ has the capacity to regulate some skeletal muscle Na_V_1.4 isoforms in a manner similar to the cardiac isoform Na_V_1.5 [14], [17], [18], [24], [32], provided the Na_V_1.4 channel isoforms have proper residues in the EFL sequence. For example, channels with the rNa_V_1.4 EFL (Figure 6A) or engineered mutant human channels with the orthologous rat residues in the EFL, hNa_V_1.4_GS/AD_, are regulated by [Ca^2+^]_i_. In contrast, channels with the native human sequence (hNa_V_1.4) or mutated residues in the EFL (rNa_V_1.4_SG/DA_) are insensitive to [Ca^2+^]_i_. This study also demonstrates that detailed regulation of inactivation of variants of Na_V_1. 4 channels by CaM over expression is determined by the composition of the EFL and the ability of intracellular Ca^2+^ to regulate gating (Figure 7). Irrespective of Na_V_1.4 channel isoform, CaM mediated regulation of Na_V_1.4 depends on the EFL residues and [Ca^2+^]_i_. The voltage dependence of inactivation gating of the skeletal muscle Na channel variants is either Ca^2+^ sensitive (rat Na_V_1. 4, hNa_V_1.4_GA/DS_) or insensitive (human Na_V_1. 4, rNa_V_1.4_DS/GA_). The Ca^2+^ sensitivity is in part determined by the amino acid sequence in the EFL. This sequence divergence is associated with differences in the baseline voltage dependence of inactivation as well as the response to CaM over expression (Figure 7).

## Conclusion

The cold aggravated myotonia mutation, hNa_V_1.4_F1705I_ in the CT of the skeletal muscle channel remote from the EFL region produces temperature-dependent slowing of current decay and significant destabilization of inactivation, and is associated with a disruption or alteration of Ca^2+^ regulation. Alteration of [Ca^2+^]_i_ sensitivity at low temperature could potentiate myotonia symptoms. These changes result in greater Na current availability at depolarized voltages, and thus prolongation of the cellular action potential which is likely to be the proximate cause of myotonia. Moreover the data suggests a mechanism by which drugs that stabilize Na current inactivation may be useful in controlling muscle symptoms. This disease causing mutation and isoform specific amino acid variation in the CT EFL provide important insights into the differences in Ca^2+^ regulation of Na_V_1 channels.

## Supporting Information

File S1 **Includes Methods, Figures S1 – S3 with legends, Table S1, and References.**
(DOCX)
