# Supplementary material for: Mechanisms of a Human Skeletal Myotonia Produced by Mutation in the C-Terminus of NaV1.4: Is Ca2+ Regulation Defective?
Source: PLoS One. 2013 Dec 6;8(12):e81063. doi: 10.1371/journal.pone.0081063 (PMC3855693; doi:10.1371/journal.pone.0081063)
Supplement: File S1 — Includes Methods, Figures S1 – S3 with legends, Table S1, and References. (DOCX) [file pone.0081063.s001.docx]

**Mechanisms of a human skeletal myotonia produced by mutation in the**

**C-terminus of Na_V_1.4: Is Ca^2+^ regulation defective?**

Subrata Biswas, Deborah A. DiSilvestre, Peihong Dong, Gordon F. Tomaselli

**Affiliations:** Department of Medicine, Division of Cardiology, Johns Hopkins University, Baltimore, Maryland, United States of America.

***Address correspondence to****: Gordon F. Tomaselli, MD., Department of Medicine, Division of Cardiology, Johns Hopkins University, 720 N Rutland Avenue, Ross 844, Baltimore, MD, USA, 21205: Tel: 410-955-2774; Fax: 410-955-7953;*

*Email: gtomasel@jhmi.edu*

**SUPPORTING INFORMATION**

**Methods**

***Measurements of free [Ca^2+^] in the patch pipette solution using Kwik-Tip Calcium Ion-selective electrode.***

Fluoride is widely used in patch pipette solutions for stable seal formation in patch clamp recordings. Fluoride can interact with a number of ions and buffers. Importantly it may bind to free Ca^2+^ reducing the level due to formation of insoluble precipitate of CaF_2_ (K_SP_ ~3.9 x 10^-11^). The solubility product of CaF_2_ is influenced by pH and other ions, as is the case with the pipette solutions used in this study. There is no reliable multi-ion simulation which can predict CaF_2_ formation and free Ca^2+^ concentration in a multi ion solution so we measured the free Ca^2+^ concentration in our pipette solutions using Ca^2+^ selective electrode.

The free [Ca^2+^] in the patch pipette solution was measured using Kwik-Tip Ion-selective electrode (ISE) according to manufacturer’s protocol. Several ions including Na^+^ have a small effect on the Ca^2+^ electrode and can interfere with the measurement but the pipette solutions were Na^+^-free. The pipette solution with nominally 0 Ca^2+^ contains (in mM): BAPTA 20, CsF 110, CsCl 20, HEPES 10, pH adjusted with CsOH to 7.35. The 0.5 µM Ca^2+^ pipette solution contains (in mM): BAPTA 5, CsF 110, CsCl 20, HEPES 10, CaCl_2_ 4; pH adjusted with CsOH to 7.35.

Standard Ca^2+^ solutions, ranging concentration 0.1 μM to 20 μM were prepared using the standard calcium solution (39 μM) and dilution buffer from the kit (Calcium Calibration Buffer Kit #1; Cat No. C3008MP; Invitrogen / Molecular Probes). 20 μM Ca^2+^ was used as reference solution. Absolute voltages were recorded for standard and pipette solutions in electrically shielded chamber placed in a Faraday cage. Measured voltages of Ca^2+^ free solution is significantly different than that of Ca^2+^ containing solutions (Table S1). The absolute voltage measurements were used calculate the concentration of Ca^2+^ in the pipette solution form the standard curve and the measured concentration of the 0.5 μM Ca^2+^ solution was 0.59 μM (Figure S1 and Table S1).

**FIGURE LEGENDS**

**Figure S1. Standard plot of [Ca^2+^].** Standard plot of [Ca^2+^] using KWIKCAL-2 (WPI, ISE) Calcium Ion Selective Electrode and Calcium Calibration Buffer Kit (Cat No.C3008MP; Invitrogen / Molecular Probes). Since Ca^2+^ ion-selective electrode can be influenced by Na^+^, this ion was not used in solutions for measurements. 0.5 μM/L patch pipette solution measures +93 mV by Ion selective electrode, which is approximately 0.59 μM.

**Figure S2. Effect of Ca^2+^ on steady-state inactivation in fluoride-free pipette solution.** The main motivation for the use of fluoride in this study is to compare our data to that previously published. There are a number of other papers that have examined the Ca^2+^ sensitivity of Na channels using intracellular fluoride and BAPTA to manipulate intracellular Ca^2+^ levels [[1](#_ENREF_1),[2](#_ENREF_2),[3](#_ENREF_3)]. Other studies [[4](#_ENREF_4),[5](#_ENREF_5)] used fluoride in the pipette solution. We believe using ionic conditions similar to previous studies is the best way to compare our data to the previously published findings. The absence of fluoride in the pipette does not change the voltage dependence or Ca^2+^ sensitivity of channel gating (Figure S2) similar to our previously published data [[6](#_ENREF_6)]. We substituted CsF with an equimolar concentration of CsCl and removed NaF from the pipette solution and recorded sodium current. Figure S2 is the plot of rat Na_V_1.4 current recordings showing effect of Ca^2+^ on steady-state inactivation in fluoride-free pipette solution.

**Figure S3. Deactivation in mutant channels.** Wild type and hNa_V_1.4_F1705I_ channel deactivation was studied in 0.5 μM Ca^2+^ and Ca^2+^-free intracellular conditions. Deactivation was assessed using tail currents elicited by a test pulse of 0.5 ms to +40 mV followed by repolarization to a family of pulses ranging from -180 to -50 mV. Current decay rates were assessed by fitting to a single exponential decay. In presence of Ca^2+^ or in Ca^2+^-free conditions τ_Deactivation_ of the wild type and myotonia mutant channels are not significantly different at more negative voltages from -100 mV to -180 mV (p < 0.05; n = 4 to 6). In the voltage range from -90 mV to -60 mV, τ_Deactivation_ of myotonia mutant in Ca^2+^ free condition is significantly greater in the wild channel compared to hNa_V_1.4_F1705I_ in the presence or absence of Ca^2+^ (p < 0.05; n = 4). However, kinetics of currents measured at more depolarizing voltages with this deactivation protocol are contaminated by inactivation.

**Figure S1. Standard plot of [Ca^2+^].**

**Figure S2. Steady-state inactivation of rNa_V_1.4 in fluoride-free pipette solution.**

**Figure S3. Deactivation of myotonia mutant channels.**

**Table S1.** Measurements of absolute voltages of Ca^2+^ solution and Patch Solution using Kwik-Tip Ion-selective electrode (ISE)

| Standard solutions from kit | mV | | |
| --- | --- | --- | --- |
|  | #1 | #2 | #3 |
| 0.1 μM CaCl_2_ | +91 | +91 | +91 |
| 1 μM CaCl_2_ | +94 | +94 | +94 |
| 10 μM CaCl_2_ | +106 | +106 | +106 |
| 20 μM CaCl_2_ | +114 | +114 | +115 |
| Pipette solutions |  |  |  |
| 0 Ca^2+^ | +52 | +51 | +52 |
| 0.5 μM Ca^2+^ | +93 | +93 | +93 |

**References**

1. Wu FF, Gordon E, Hoffman EP, Cannon SC (2005) A C-terminal skeletal muscle sodium channel mutation associated with myotonia disrupts fast inactivation. J Physiol 565: 371-380.

2. Casini S, Verkerk AO, van Borren MM, van Ginneken AC, Veldkamp MW, et al. (2009) Intracellular calcium modulation of voltage-gated sodium channels in ventricular myocytes. Cardiovasc Res 81: 72-81.

3. Wingo TL, Shah VN, Anderson ME, Lybrand TP, Chazin WJ, et al. (2004) An EF-hand in the sodium channel couples intracellular calcium to cardiac excitability. Nat Struct Mol Biol 11: 219-225.

4. Tan HL, Kupershmidt S, Zhang R, Stepanovic S, Roden DM, et al. (2002) A calcium sensor in the sodium channel modulates cardiac excitability. Nature 415: 442-447.

5. Young KA, Caldwell JH (2005) Modulation of skeletal and cardiac voltage-gated sodium channels by calmodulin. J Physiol 565: 349-370.

6. Biswas S, Deschenes I, Disilvestre D, Tian Y, Halperin VL, et al. (2008) Calmodulin regulation of Nav1.4 current: role of binding to the carboxyl terminus. J Gen Physiol 131: 197-209.
